# A phenotypic, transcriptional and TCR Vβ repertoire signature of CD8+ T cells define a population at-risk of long-term kidney graft dysfunction

**DOI:** 10.1186/1479-5876-10-S3-P59

**Published:** 2012-11-28

**Authors:** Nicolas Degauque, Françoise Boeffard, Annaick Pallier, Richard Danger, Magali Giral, Jacques Dantal, Yohann Foucher, Cécile Guillot-Gueguen, Jean-Paul Soulillou, Sophie Brouard

**Affiliations:** 1 INSERM, UMR 1064, Nantes, France; 2 CHU de Nantes, ITUN, Nantes, France; 3 Université de Nantes, Faculté de Médecine, Nantes, France; 4 Université de Nantes, EA 4275, Nantes, France

## Introduction

The biological mechanisms leading to chronic antibody-mediated rejection (CAMR), a major cause of late graft failure following kidney transplantation, are still poorly defined. Although anti-donor HLA antibodies are commonly associated with poor graft outcome; less attention had been paid to other players of the adaptive immune system.

## Aim

We took advantage of a large cohort of 133 selected patients (112 patients remaining stable in time and 21 with kidney dysfunction over 6 years) to question the factors that may influence graft outcome.

## Results

We show that T cell monitoring, and especially CD8 TCR repertoire alterations, may allow identifying patients at risk of graft dysfunction. As compared to patients without TCR Vβ repertoire alterations, patients with an altered TCR Vβ repertoire at the inclusion have a 2.1 fold higher risk of graft dysfunction during their follow-up. The Vβ repertoire alteration occurs years before the appearance of de novo anti-HLA antibodies.

Moreover, these patients with an altered TCR repertoire exhibit an increase in effector memory CD45RA^-^CD197^-^CD8^+^ T cells with an accumulation of differentiated (CD28^low^) CD8 T cells. Finally, a specific CD8 gene expression pattern composed of 92 genes related to CD8 T cell function and phenotype can discriminate these patients from patients with lesions of CAMR.

## Conclusions

Monitoring the TCR Vβ repertoire of circulating CD8 T cells may help to improve the identification of at-risk patients before the detection of HLA antibodies. Besides offering a new tool for monitoring patients, our data shed new light on the status of T cell immunity in long-term graft outcome.

